# Perspectives on influential factors for the adoption of circular economy practices

**DOI:** 10.1007/s43615-026-01061-4

**Published:** 2026-09-02

**Authors:** Cleber J.C. Dutra

**Affiliations:** 1 Institute of Economics, Finance, and Management, Jagiellonian University in Krakow, Krakow, Poland; 2 Sustainability and Innovation Research Group, School of Management / UFRGS, Porto Alegre, Brazil

**Keywords:** Circular economy, Sustainability, Sustainable practices, Influential factors, Fields of influence

## Abstract

This study aims at contributing to improve understandings of influences that factors may exert on processes of adoption of circular economy (CE) practices by diverse types of organisations. Its propositions offer perspectives structured from categories identified as suitably grouping these factors. Each category characterises areas of influence, according to kinds of contribution, impact, or effect their factors exert. Seen from analytical dimensions used for the typification, the categories also characterise levels and fields, expanding the capacity for understanding their influence through dimensional meaningful perspectives. Resulting from applying strictly inductive and Complexity-based approaches, findings unveil complex perspectives on issues of CE adoption processes. Thus, the typification of elements, relationships, dimensions, and inter-relational dynamics involved in CE phenomena can assist in the development of conceptual propositions that enable theoretical progress in the field of CE studies, which benefits strategic, managerial, and assessment roles in the implementation of CE practices.

## Introduction

A disquieting registered declining course of the world circularity [1–3] inspires concern and reflections on potential reasons for this downturn and apparent lack of success of globally widespread collective efforts aimed at promoting Circular Economy as a viable path towards sustainability. Drawing particular attention to a decade of major commitments made by the European Union to the formulation, issuing, and enforcement of Circular Economy (CE) strategies, plans, and regulatory instruments [e.g. 4–7], sizeable achievements were to be expected. While assessments rank the Netherlands as a global frontrunner towards circularity (based on its circularity metric of 24.5%) [8], most countries in Europe are still struggling with their CE progress, and the 75% still to be achieved by the Netherlands and fulfil its target of full circularity by 2050 indicate evident challenges ahead.

Assessments are instrumental in guiding strategic measures towards successful accomplishments in CE implementation. A recent comprehensive review [9] examining the scientific progress in evaluating CE performance of European countries analyses severe limitations faced by researchers, despite availing of Eurostat’s comprehensive database and the leading-edge advanced means provided by it. This constraint adds hurdles for a region where much has been invested in CE regulations and enforcement, but also in extensive infrastructure, normative developments, and corresponding rollouts of procedures to measure, register, process, and publish CE-related indexes. Hence, complex challenges, also in this aspect, demand vital attention from all involved parties engaged in replacing linear patterns of production and consumption by circular ones.

Further fundamental challenging conundrums defying progress in research, theorising, explanatory capacities, abilities linked to conceptualising, planning, and managing functions regarding CE issues -- as a whole set of needed competences to succeed in this endeavour -- are complex inherent attributes, processes, and dynamics involved in its nature. Among the pioneering studies discerning CE as an emerging ample field of multidisciplinary research, and valuing the importance of framing systematised avenues for categorising, organising, and assessing progress of its constitutive streams, Korhonen et al. [10] performed a meticulous analysis of the field. Besides proposing Complexity as a central dimension to examine and assess the progress of its scientific evolution, they contend that -- in view of the verified paucity of studies in more complex aspects of CE -- an increase in research focused on topics of higher level of Complexity would be necessary, in order to promote the paradigmatic shift envisioned for this field.

Recent conceptual contributions have been demonstrating the benefits of more complex perspectives for improving the understanding of advanced aspects of CE issues. A systematisation of barriers, as factors identified in a literature review conducted by Sopjani et al. [11] hindering the transition towards circularity, helps improve the comprehension of multiple aspects related to contexts, strategies, levels, research approaches, and understandings associated with inhibitory influences captured by diverse studies. Such multidimensionality of the systematic examination of negative roles exerted by barriers also demonstrates the complexity involved, especially when the authors recommend further investigations into the interactions between these factors and contributions to clarify the understanding of their complex implications. In search of a privileged perspective that allowed the understanding of the mechanisms underlying transition processes from linear to circular regimes of production and consumption, Blomsma et al. [12] propose the combination of views, elements, phases, functions, and dynamics from three conceptual models/approaches (S curves; Panarchy; and TIS - Technological innovation systems) to build a framework and the concept of Circular Disruption, which help explain how the transition occurs and how to accelerate it. Again, this multidimensional view proposed by their contribution adds a highly complex perspective to the study of CE transition processes, benefiting considerably the comprehension of how a multitude of factors described along the phases of Circular Disruption processes could favour the transition. Also having in view the importance of speeding up the CE transition and of overcoming barriers hindering it, Iacovidou et al. [13] expand their analyses of resource recovery systems, proposing the structure of a system of sub-systems, three lenses for analysis of dynamics (related to: production-consumption-management; processes-actors-values; and five levels of information), and how these components of their framework perform roles in the analysed processes. By examining in detail several aspects and occurrences involving production and consumption practices and implications for (in)efficient attempts to close resource loops, their expositions cover numerous factors involved in the processes, as well as their complex interactions and influences in diverse ways. Relationships regarding their framework’s (multiple) elements and the profusion of dynamics indicated throughout their explanations attest to the complexity of the perspectives they propose, demonstrating how these complex views improve significantly the understanding of the processes.

Therefore, as the preceding expository points introduced, part of the reasons for the apparent unsuccessful performance of attempts to implement CE practices, even when plenty has been invested in this purpose, is essentially connected to the complex nature of a circularity paradigm. Appreciating this inevitable actuality, Kirchherr et al. [14] reflect on the risk that, due to exceedingly complexity involved in CE implementation, practitioners and academics may turn away from the field. In order to tackle this factual dilemma, dealing with such inherent complexity constitutes a task that, as reflected by Tudor and Dutra [15], requires a set of perspectives from theoretical and practical standpoints, which might be amplified to conceive: ways of exploiting complexity approaches proposed by Bicket [16]; advanced business models from sophisticated viewpoints explained by Henry and Kirchherr, both theoretically [17] and practically [18]; or adaptions of complex economic approaches to enhance sustainability outcomes in regional planning, as examined by Monteiro and Dutra [19]. Congruent with the three conceptual contributions examined in the previous paragraph, these possibilities of applying complex perspectives to CE phenomena help build expanded competences that assist us in overcoming the risk against which Kirchherr et al. [14] caution.

The present study draws intrinsically upon this view of the benefits that applying more complex lenses can bring to improve the understanding of CE adoption processes, and explores this approach by examining in-depth the knowledge from evidence provided by varied CE experienced specialists. Similarly to the cited studies, in which the examination of barriers [11] and drivers & barriers [13] to implementation processes was a focal component, the focus was directed to the profiling of factors and their influences on processes. Opting for a strictly inductive process of enquiry, the procedures were conducted by adopting a -- as much as possible -- fresh view of events, situations, and their circumstances, suspending at this stage of the research process the theoretical contributions from the literature. Hence, the analyses conducted in the study did not resort to literature reviews and corresponding theoretical underpinning as a support. This approach favours originality and openness to new ways of seeing adoption of CE practices.

CE studies employing similar inductive methodologies progress within this literature area. Although substantial contributions to the scientific knowledge on barriers and drivers to CE adoption have been provided by research based on grounded theory (GT) procedures [e.g. 20–26] the present study differentiates itself from this segment of the literature by:

- reinstating values of original propositions by Glaser & Strauss [27]; particularly regarding avoidance of the employment of pre-existing theoretical contributions in the identification of factors, and proposition of categories;
- delaying intentionally any comparisons of findings with pre-existing concepts (from the literature) until reasonable degrees of maturity are reached in the theoretical propositions being developed (as more recently argued by Glaser [28, 29]);
- concentrating intensively on the examination of potential interactions between/among factors and of their influences;
- proposing an integrated framework encompassing all categories and their factors, which serves as a heuristic tool to reflect on their potential influences on the CE adoption;
- not being restricted to one region or one country; and
- having analyses not limited to industrial sectors.

Additionally, since analyses of complex phenomena related to CE issues that are underpinned by Edgar Morin’s concepts virtually lack in the literature, this study adds Morin’s analytical views to its unique characteristics just mentioned, awarding it the character of being innovative and entirely original.

Along with contributing towards amplifying views (and, therefore, competences) for analysis of aspects related to CE adoption, exploring complex features of influential factors, the areas of influence categorised in this study serve as references for the design and planning of interventions aimed at implementing CE. Among the limitations identified in research on the assessment of CE performance [9], indicators are considered inadequate to express several features of circularity, and recommendations made include the consideration of indexes that represent such neglected aspects. Types of influence characterised by the factors examined in this paper may provide prospective parameters to be considered. Strategising, managing, and assessing CE processes might benefit from the outcomes achieved so far in the present study.

Organised in the three subsequent sections, the contents of the paper develop, explain, deepen, and discuss points and notions outlined in this Introduction. Section  Methodological underpinning and approaches describes particularities of the methodological underpinning and procedures of the study, clarifying choices made and their reasons. Results and their current contributions, at this stage of the study, to the aims of this research initiative are presented in Sect.  Current results and analytical perspectives . The last section comments on current accomplishments, findings and insights, potential further developments and implications, as well as following progress projected in the next phases to be concluded for this research.

## Methodological underpinning and approaches

Aspiring to unearth views of aspects that might be novel or unexplored for the understanding of processes of adoption of CE practices, the study’s research design opted for returning to seminal propositions for building theoretical explanatory structures from empirical qualitative analysis of raw data [27]. By assuming a thorough open-minded posture, a full receptivity to novelty was prioritised as an aim to support the development of the study’s propositions.

### Research paradigm alignment

Processes of adoption of CE practices by organisations, considering all stakeholders (internal and external) involved in the whole network of actors participating in these processes, are highly complex phenomena, especially when all dimensions (e.g. technological, social, environmental, institutional, economic, cultural, financial, behavioural, political, managerial, among others) that add an explanatory view to a more comprehensive understanding of them are taken into account for the analysis of a complete set of organisations engaged in the process. Aligned with this premise, this study’s epistemological paradigm is underpinned by Edgar Morin’s foundations of Complexity [30], using such wider capacity for the comprehension of the intricacies of these multidimensional processes to improve the possibility of proposing perspectives that help better understand them.

### Methodological choices and reasoning

Based on the assumption that even the most experienced expert in the topic of this research would have a *partial view* of these complex processes, and no single individual comprehends, in its entirety, the whole phenomenon of adoption of CE practices by an organisation, this research opted for collecting and accumulating views of the adoption processes from diverse CE experts and examining their partial understanding as elements to build a more comprehensive heuristic tool that could offer ways to integrate expanded perspectives on these processes.

This approach bears similarities with conceptual foundations for building theoretical propositions set by Glaser & Strauss [27], through strict inductive cognitive processes of studying social phenomena, which inspired the procedures followed in this study. For guiding the abstraction phase of formulating concepts, notions regarding the dimensions devised by Strauss & Corbin [31] were considered.

### Unit of analysis, sources selection and role of participants, and theoretical abstraction goals and paths

Aiming at improving understandings of (i) *influences* that factors have on processes of CE adoption, and (ii) *interactions/relationships* these factors and their influences have throughout these processes, analyses depart from *factors*. Therefore, in order to examine (i) influences, and (ii) interactions/relationships, the focal element for analyses is a *factor*, which is characterised as *any element that has potential kinds of contribution, impact, or effect (favouring and/or hindering) a CE adoption process*.

Sources of evidence that provided minimal enough contents to perform an analytical procedure, thus specified as *unit of analysis*, are *any accounts* describing any *potential kinds of contribution, impact, or effect* which affect *(favouring and/or hindering)* processes of CE adoption, according to views and experiences of persons engaged in CE practices. All types of data mentioned in the subsection Data collection, analysis and processing, and formulation of propositions contribute as source, sometimes being a *single sentence* said by a participant in a meeting of experts, written down as a single note by a member of the research team. Countless events like this offered abundant, rich contents to the analyses during the period of data collection. Participants in the collection of evidence were persons experienced in CE issues, who contributed sometimes one single sentence (e.g. in a lecture, conference presentation, observed dialog, experts’ meeting, short conversation, among other events) or several relevant contents (in similar events and in-depth interviews).

Once an analysed *account* provided evidence that enabled the identification of a *factor*, this factor’s *influence*, and further information supporting the understanding of this factor’s *interactions/relationships* which affect processes of CE adoption, these pieces of information become contents enabling *conceptualisations* aimed at in this study as components of the *theoretical abstractions*. Their goals were: the characterisation of the influences; the typification of *contributions, impacts, and/or effects*; the identification of potential *categories* (according to the types observed); the observation of *interactions* and *relationships *(between/among *factors*, *influences*, and *categories*); the identification of potential dimensions framing areas/fields and levels of influence characterised by the categories and their relationships; the organisation of all these components in clear structures, which served as potential frameworks. Alongside these analytical goals, which also constitute directions to be followed, procedural paths were outlined, leading the analytical process towards the achievement of the final outcome of the theoretical abstraction, the proposition of a framework.

### Data collection, analysis and processing, and formulation of propositions

Data collected followed the workplan of the research project mentioned (see Funding) conducted for two years (2023–2025), of primary and secondary sources, consisting of interviews, notes from observations, researcher field notes, notes from conferences and public events, reports, and other public documents. Secondary sources of evidence concentrated on publications describing the implementation of circular economy programmes in the three countries focused on in the project, and primary sources -- at the stage of the analyses performed which correspond to the results presented herein --, were collected mainly in Italy and Ireland and marginally in Poland. Events (as the ones described in the subsection Unit of analysis, sources selection and role of participants, and theoretical abstraction goals and paths), from which data were collected as contributions provided by CE experts, counted with participants from all over the world (in particular, participants expressed views acquired mostly from their CE experiences in: Australia, Austria, Brazil, Canada, Colombia, England, Germany, Ghana, India, Ireland, Italy, Kenya, Netherlands, New Zealand, Poland, Scotland, South Africa, Ukraine, and Wales), and the sources of evidence were, therefore, not limited to views from European countries.

Factors were identified through content analyses of notes and comments on processes of adoption of CE practices in different data collection procedures, sometimes being clarified through further dialog with participants in the events observed and interviews. For the identification of factors, attention was given to any elements characterised as specified in the subsection Unit of analysis, sources selection and role of participants, and theoretical abstraction goals and paths. Characteristics identified from the examination of factors’ influences, and their interactions and relationships provided meanings that supported the proposition of areas/fields of influence, constituting potential categories, which could be eventually merged as one category (as the ones proposed in the Framework), considering similarities among their meanings-related types of influence. Influences observed between/among factors grouped in specific categories, and in their chain of effects on processes of adoption of CE practices indicated flows of influence that should be depicted in the structures proposed for the Framework. Drafts of such proposed structures were gradually improved to better reflect the findings from all analytical procedures.

According to the inspiration in concepts by Glaser & Strauss [27], analyses respected an absolutely open-minded posture towards possible factors. The same posture was observed in the identification of possible categories to which the factors could belong to, as well as the relationships between/among factors. Regarding these relationships, in order to expand their understanding and acquire a more comprehensive view of their interactions, properties, etc., their examination observed Morin’s complex principles -- as: the dialogic (reconciling contradictions); the recursive (effects becoming causes in non-linear feedback loops); the hologrammatic; interconnectedness; uncertainty and incompleteness; multidimensionality of knowledge; among others [30] -- without the purpose of classifying them under such principles.

The identification and typification of areas and fields of influence to which the categories could be associated with followed the same sources of guidance and posture. Considering the dimensions to be identified [31], they were proposed based on their potential to facilitate an understanding of processes of adoption of the CE practices. The draftings of proposition of these relationships and their structure observed recursive dynamics.

### Data availability and participant confidentiality

The sources of evidence (particularly interview transcripts generated, notes from observations, researcher field notes, notes from conferences and public events) analysed for this study are not available in a public repository due to constraints regarding protection of participants. These documents contain sensitive and potentially identifying information and, although participants provided consent for using their contributions following anonymising procedures, such procedures would not prevent the risk of participants being identified. Public release of full transcripts and notes would create an unacceptable risk of re-identification because notes and interview contents include detailed contextual information (references to the organisation/employer, workplace events, small-group roles, etc.). Most of participants carry their roles at public organisations/institutions and having almost unique functions and engagements in their area of expertise. Our research team fully support FAIR principles, however, for this study, public sharing of the raw sources would violate explicit confidentiality promises and could realistically lead to participant identification.

## Current results and analytical perspectives

**Fig. 1:**
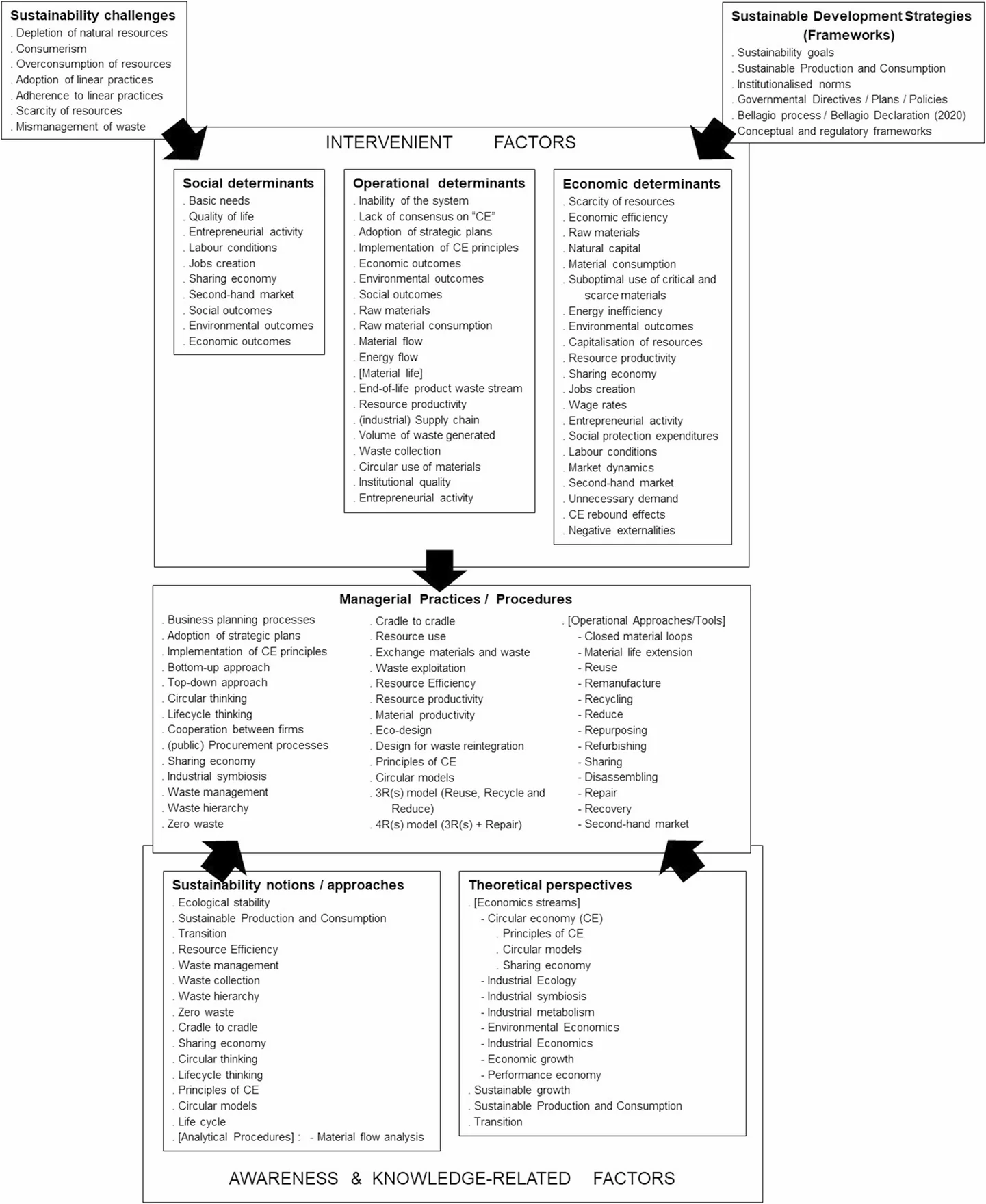
Influential factors on circular economy management practices organised in categorised areas

Analyses of all sources collected yielded a massive amount of factors identified as having influence on processes of adoption of CE practices, according to the methodological characterisation of a *factor*. Examining features of their influence (and similarities among these) enabled the merging of potential meanings of their effects/impacts on, and contributions to (favouring and/or hindering) the processes into certain potential types. Similarities among such meanings-related types of influence enabled the merging of groups of similar types into the proposed categories. Aiming at facilitating the understanding of this study’s findings (at the current stage achieved for the proposed theoretical propositions), it seemed better to start this section with the resulting representation of the study’s outcomes, in the form of a framework, and dedicate the following subsections to describe the gradual developments that led to this framework. Thus, the grouping of factors into the identified categories is shown as the study’s current Framework in Fig. 1. Further explanations about theoretical features embedded in the framework, and comments on the perspectives resulting from the analyses receive proper attention in the subsequent subsections.

It is essential, for the understanding of the results presented herein, to clarify that the aim of the present study and its propositions is not to exhaust all possibilities of influential factors and their fields of influence. Hence, neither the lists of factors nor their proposed categories are comprehensive. Numerous (additional) factors can be added to the proposed categories, as well as further potential areas and fields of influence might complement the conceptual structure of relationships described here.

Also, it should not be surprising that the factors exhibited in the Framework are commonly found (and discussed) in the literature on drivers and barriers. Accumulated research, both, theoretical and empirical, on this topic -- as hinted at by the mention of the very few references cited herein --, reveals views from scholars and practitioners of factors favouring and hindering processes of CE adoption, which should not likely differ from the ones presented here. Nevertheless, interactions / relationships examined in the influential factors’ features have been scarcely investigated, and, in the case of GT-underpinned studies, particularly limited to local, regional, and/or national CE issues / phenomena. GT approaches enable deeper reflections and levels of abstraction in in-depth examinations, and this choice assisted the present preliminary investigation of complex interactions related to factors’ influences on CE adoption processes. Therefore, since this study’s ultimate aim does not focus on the factors, but on relationships observed between/among them and their influences, as well as on potential dynamics identified in the categories’ interactions, this is what should be expected from aspects discussed in this section’s analyses and preliminarily proposed framework layout.

For those who are familiar with Edgar Morin’s work on Complexity, it also should be expected that -- in order to follow Morin’s approach and be true to his epistemological stream of thought -- the analyses developed here shall emerge through reflections drawn from descriptions of the observed findings and described phenomena, based on which the complex properties found in the research results can be revealed [32, 30]. According to this feature of Morin’s explanatory pattern, discussions are introduced in a predominantly descriptive process, laying the bases for new perspectives to be presented. Additionally, as a mechanism to highlight recursivity and recursive properties of the observed dynamics, this epistemological pattern tends to be seen as repetitive, despite being rather a process of closing loops and spirals of developing knowledge.

Thus, in congruence with Morin’s work, besides seeking coherence in the attempt made of integrating this study’s research paradigm with its methodological choices for a knowledge development which can be considered as in its exploratory stage, the results assembled in this section express yields of a long period of examination of a very large amount of data through an innovative approach, though fairly complex. The character of the study’s contributions reflects the character of its complex efforts.

### Framework structure and rationale

The outcomes of this study show possibilities of categorising factors into some areas / fields of influence, which open perspectives for the understanding of processes leading to the adoption of CE practices by the management of diverse organisations (Fig. 1). These areas represent dimensional views of types of influence, depending on how they contribute to the adoption of CE concepts, approaches, requirements, strategies, and further aims for CE principles.

The eight categories composing the Framework are positioned in a way that, examining favouring contributions exerted by the factors they comprise, their (positive) influences lead to the adoption of CE practices by an organisation, as represented by the category Managerial Practices / Procedures (centre of the figure). The influences observed at the top of the structure -- from the two categories, Sustainability Challenges, and Sustainable Development Strategies (Frameworks) -- could be seen as equivalent to pressures on involved actors / stakeholders in a way that would favour the processes of CE adoption. At the bottom of the Framework structure, both categories would assist an organisation’s managers in adopting CE practices by contributing to build capacity for managing those practices / procedures. The categories proposed as Determinants (Social, Operational, Economic) would modulate, variously, influences on the adoption processes, adding particularities, specificities, and/or interpretative aspects to a process, but also, depending on the examined cases, having a more independent / autonomous influence on the adoption. As a general overview, these logical considerations guided choices for structuring the Framework.

Each category expresses a type of meaning and the name chosen for the category indicates the role the factors grouped into it exert, as a type of influence. For instance, factors grouped into Sustainable Development (SD) Strategies (or Frameworks) have normative or regulatory roles; Sustainability Notions / Approaches group factors that represent a concept that applies to circularity aspects, principles, processes, aims, and assists in its achievement; while factors included in Sustainability Challenges raise concerns about and characterise threats to sustainable standards or to their achievement. Further clarification of these meanings and roles the factors exert, as influence on processes, are presented in the following subsections, where examples of influential contributions of factors (often of multiple types) are outlined.

### Abstracting sense from factors

The identification of factors (following their proposed definition) provided multitudinous elements having any contribution (positive, when favouring the adoption process; negative, when hindering it) to the processes analysed. For instance, this broad definition allowed freedom to the participants / interviewees to comment on any elements -- and in all dimensions mentioned in the subsection Research paradigm alignment -- that, according to their experience, influenced the process of adoption of CE-related practices. However, to list all factors found would bring no clarification to this paper, considering that the aim of this study was to condense its results into a concise theoretical representation of its findings. During the analyses, it was important to abstract the types of influence of each factor by associating a meaning to each type. The collection of meanings provided a procedure to identify dimensional views of types of influence, which assisted in the process of categorising the areas of influence. After this phase, it became possible to see if the meanings perceived for each new factor identified was represented in the compilation of types developed. Through this process of theoretical saturation, following the steps described at the introduction of this section, the categories seen in the Framework were proposed.

Therefore, the outcomes of these procedures should allow further analyses in CE studies to examine if factors not listed in the Framework could also belong to the dimensions of influence proposed herein. The accomplishment of such goal would indicate the theoretical value of the Framework, assisting the understanding of processes of adoption without needing to list all factors identified in this study’s analyses. Hence, the factors listed in the categories (Fig. 1) serve the purpose of helping to comprehend what each area of influence contributes to the adoption process, being thus examples which facilitate analytical processes with the support of the Framework.

### Categories as areas / fields of influence and perspectives of levels

The categories proposed represent analytical dimensions that assist in the understanding of adoption processes, as areas of influence. Further phases of abstraction in this study’s analyses provided views of the processes which suggested the possibility of correlating these areas of influence, thus proposing a higher analytical dimension for the understanding of the processes. This attempt resulted in the grouping of the categories into clusters, as illustrated in the Framework as fields of influence.

In the current stage of this study, the categories Sustainability Notions / Approaches and Theoretical Perspectives are clustered together in a field proposed as Awareness & Knowledge-Related Factors. Considering the contributions that, for example, CE notions and CE theoretical foundations bring to build competences necessary for the management of CE practices adopted by organisations, their areas of influence can be seen as correlated. In the analyses of meanings identified, Notions are perceived as more widespread among CE practitioners, while Theoretical Perspectives can be considered closely associated with knowledge developed, shared, and provided by scholars. Mechanisms of transfer of both these types of knowledge can coincide, or vary, depending on the degree of formal / informal training or educational processes of acquisition.

Similarly, the categories proposed as Determinants (according to their types of influence explained in subsection Framework structure and rationale) share correlated influential roles, suggesting the possibility of clustering them into the field Intervenient Factors and having, in many cases, roles of modulating influences. This additional analytical dimension does not preclude, however, the examination of their factors as exerting direct influence on the adoption of CE practices. Explanations related to these categories in the following subsection clarify further this aspect.

Analysing the types of influence according to more direct or distant kind of contribution, impact, or effect they bring to the processes could indicate potential similarities with perspectives of levels of dimensional understanding. For instance, the two categories on the top of Fig. 1 can be seen as at a macro level, while the Managerial Practices category groups factors closer to a micro view. The three categories identified as Determinants, considering the perspective of having an intervenient role, can be seen to be at an intermediate level. Again, the suggestion of levels as an additional dimension of analysis, aimed at expanding the understanding of processes, should not prevent the examination of aspects related to the influence of factors from being analysed by omitting such additional perspective.

### Perspectives on complex influences exerted by factors

By using the Framework as a support, the presence (or absence) of the factors helps the understanding of grounds favouring (or lacking in) the performance of CE practices. In this sense, employing the factors -- and more accurately, the type of their influence as typified by the category they belong to -- would serve to improve the understanding of the processes involved in the adoption and establishment of such practices. In other words, the presence of a factor (or its type of influence) favouring the adoption, or the absence of factors (or their type of influence) hindering it, aids an understanding of the issues contributing to the adoption/establishment of a CE practice within organisations.

Thus, the two categories at the top (Fig. 1), Challenges and well-enforced CE regulations and policies (like in the category SD Frameworks) can exert a critical influence on the adoption of CE practices in the view of participants in this study. The same considerations are given to the influence of factors grouped in the two categories at the bottom (Fig. 1), given that managers and staff who acquired knowledge (through educational or training processes or practical experiences) generally achieve better performance in CE practices in their organisations. Although these four categories exert critical influence on processes, it became evident from the analyses, there would be limited value trying to establish a weighting procedure to determine which factors have higher importance or larger influence on issues related to the CE adoption. Each stakeholder involved in a process has unique perceptions of values associated with the meaning of each influential factor. The same factors might have different influences on different stakeholders, and a particular factor can have different influences on the same stakeholder, depending on the circumstances in which this stakeholder may be influenced by this factor. Therefore, in any given circumstances a factor might have more or less importance and strength, regarding its influence on the behaviour of a certain stakeholder. Indeed, each stakeholder is influenced by sets of factors in varied circumstances, and the combination of all influences exerts different effects in different circumstances.

Therefore, the analyses of cases and processes entail, evidently, the examination of as much as possible identifiable factors (types of their influence) and of the combination of contributions these sets of elements bring to the understanding of the cases / processes. Weaving together these dimensional views/understandings is the challenge that complex ways of thinking assist us to overcome.

And so starts the endeavour of complexifying analyses of CE adoption processes aimed at expanding the comprehension of their intricacies. The detailed analysis of each factor requires the examination of how their influence exerts positive or negative effects, or both simultaneously, which is frequently observed. For instance, while a large Volume of Waste Generated, as an influential factor (seen in the Operational Determinants category) causes negative environmental impacts and demands further consumption of energy and financial resources to be proper technologically processed -- effects that represent patterns in opposition to circularity ideals --, it may stimulate, concomitantly, the adoption of CE procedures aimed at minimising these negative impacts. Conversely, economic prosperity (translated in the Framework as a factor identified within Economic Outcomes) is emphasised by participants interviewed as a positive contribution to the implementation of CE programmes, due to the funding offered to stimulate innovation and developments towards circular business models. And results can be verified, in terms of CE practices adopted, being influenced by this favouring factor. At the same time, participants also argue opposite influences exerted by this factor, explaining that it inhibits efforts aimed at resource efficiency by companies still not convinced of the financial benefits from circular practices. Further views defend the positive influences of prosperity on higher levels of environmental awareness and sustainability concerns in certain European countries, corresponding to enhanced readiness to adopt CE practices by organisations and citizens. However, it is also argued that, in these societies, the generation of wastes is significantly higher, concomitantly with higher consumerism and the disposal of products that are often still new and/or functional. Such contradictory effects demonstrate concomitant opposed influences observed in the analyses of factors.

Further challenges of analysing adoption processes by examining the factors pertain to the urge to assess multiple (diverse) influences exerted by each factor, considering the roles several factors have in different dimensional facets of a process. Participants’ remarks mentioned, revealing prosperity’s complex influences, refer to this issue. Taking such analytical aspect into account, it becomes understandable that the identified factors can be analysed for their influence in more categories.

This potential extension can be illustrated with the analyses of the influences of the factor Scarcity of Resources, as a Sustainability Challenge (category on the top of Fig. 1). Scarcity encourages the development of approaches favouring more efficient (circular) ways of using resources -- type of influence outlined for the Challenges category in the second paragraph of this subsection, and also in the second paragraph of subsection Framework structure and rationale. Therefore, the effects of this influence can be clearly verified in the SD Strategies and Frameworks, when many policies and regulatory instruments have been proposed and issued based on the urge to promote practices that provide more careful ways to protect, save, and optimise the use of resources. Besides this clear influence on the SD Frameworks category, the same type of influence can be confirmed in the analyses of the category Sustainability Notions / Approaches (at the bottom of Fig. 1), by distinguishing the multitude of concepts developed, inspired by the problem posed by Scarcity, and available among approaches, as exemplified in the list of factors included in this category. Scarcity is also included among the factors in the Economic Determinants category, and can be analysed for various influences within this analytical dimensional view, from competitive behaviours (not always associated with positive ones), modulated by this factor, to its Intervenient role in the adoption, by an organisation’s managers, of Practices (category in the centre of Fig. 1) that help them tackle this significant issue. In this particular aspect, the influences of one factor (like Scarcity) on all categories, and of all categories (as the ones referred to in this paragraph) on adoption processes serve as a support to understand the process of adoption of CE procedures by managers. Thus, the recursive interactions of all these influences of one factor -- in (and between) (or among) the categories mentioned -- expand the capacity for examining (and comprehending) the adoption processes.

As a possibility to extend this analysis to a deeper level of reflection, Scarcity is a seminal concept that inspired the development of Economic theories, and, thus, its influence is also confirmed in the analyses of contributions to be considered for the Theoretical Perspectives category (at the bottom of Fig. 1). Seen from this viewpoint, the influences of all theoretical streams (the ones listed in this category serve as examples), in which the role of Scarcity contributed to the development of CE concepts, could be examined in all the other categories of the Framework. Other possibilities of extending this analytical exercise could include the direct influence of the pressure exerted by Scarcity (as a factor) on Social Determinants, considering how it affects all factors belonging to this category, and in many dimensions (behavioural, geographical, economic, cultural, political, etc.). The same exercise can offer possibilities of expanding the comprehension of processes related to CE practices, when applied to the Operational Determinants category.

Such in-depth examination of the influences of factors entails, therefore, employing all multidisciplinary perspectives and dimensions related to sustainability, expanding the capacity of comprehension of the phenomena analysed. Factors like Environmental Outcomes (which exert influence in all Determinants categories) affect, both positively and negatively, social aspects, for their effects (on the quality of air, water, and soil) impact health from different ways, determining Quality of Life (included as a factor in the Social Determinants category), which, in turn, has significant influences on Economic (as a Determinants category) issues, in multiple senses. Necessary measures to deal with negative Environmental Outcomes depend on technological considerations (influencing Operational aspects, also to be analysed as Determinants), which have strong and multiple Economic impacts (requiring analyses in this corresponding Determinants category). This illustrative exercise exemplifies the complex examination to be expected for all factors included in the categories proposed.

As a heuristic tool (subsection Methodological choices and reasoning), being rather flexible than prescriptive, the Framework suits adequately, in its current provisional configuration, the purpose of assisting in these exercises that offer better understanding of complex aspects of the phenomena it is aimed at. Therefore, considering the intention of providing perspectives that expand the comprehension of CE adoption processes, of their complexity, and the competences to deal with such challenge, this use of the Framework represents a step forward.

### Challenges of establishing competences to understand processes

The support offered by the Framework, its categories, and their influential factors for the analysis of processes of CE adoption by organisations contributes to improvements in studies of cases and CE-related phenomena, providing analytical perspectives to be explored when looking at possibilities to understand how circularity is introduced and established in these organisations’ practices. The explanations, propositions, and examples presented in this section show potential ways to explore such possibilities by employing views unlocked by the indicated perspectives. As suggested in the previous subsection, these views unfold depending on how far and how deep we intend to expand/extend the analyses of (and reflections on) multidimensional aspects, dynamics, and implications of the processes investigated. Due to the evident complexity of phenomena, concepts, and expectations related to CE aspirations, more comprehensive views of processes taken as object of study/analysis demand corresponding comprehensive scopes and in-depth examination of all relevant elements that contribute to the comprehension of these processes. Therefore, corresponding all-encompassing (or less limited) analytical tools, techniques, and approaches might be worth attention and being considered in CE research.

Evidently, satisfactory performance and successful achievements from the employment of such more advanced analytical means require corresponding advanced competences in conducting the examinations aimed at. Reflecting on the fact that our scientific education, traditional guidance, and regular practice prioritise simplifications, reduction of variables, emphasising main components of analyses, eliminating as much aspects as possible that contribute less to justify linear causality explanations, the proposed analytical expeditions sound unfamiliar (and even antagonistic) to current routines of research. Moreover, they represent uncharted territory for investigative initiatives, to which the corresponding competences might be lacking. Such challenges regarding the application of the presented propositions and the questions about how to develop (and establish) necessary competences to employ these propositions constitute a puzzle for sceptics about Complexity streams of thought, which could be answered by attempts to gradually exercise the use of perspectives as the ones suggested in this study. The illustrative notes and brief expositions presented in this section serve as glimpses of what is possible to envision, in terms of understanding of adoption processes, by experimenting the lenses offered through the proposed perspectives for the observation of complex features identified in this study’s conducted analyses.

## Resultant inferences and ensuing prospects

The proposed Framework suggests the outputs and outcomes that could be achieved by adopting methodological choices and approaches based on the combination of strictly inductive processes of analysis and the unusual way of reasoning offered by Edgar Morin’s foundations of Complexity. Its provisional character portrays current theoretical propositions underpinned by perspectives which analyses of participants’ views (as CE experts) provided. Despite the modest accomplishments presented in this paper, the achieved outcomes might inspire further research employing similar methodological options.

The areas / fields of influence from the analyses offer possibilities for conceptual dimensions that underlie further theoretical formulations. Their progressive development will improve understandings of complex facets inherent in processes of CE implementation. These aspects affecting adoption processes impact research and applied knowledge, from the phases of designing strategies for interventions (e.g. planning components of their deployment, managing their enactment/rollout), to managerial steps regarding steering their developments, including the assessment of their outcomes.

For instance, according to participants of this study, who emphasised the importance of regulations in the whole process of CE adoption, careful attention should be given to lacking comprehensive view of the influences these regulations exert, particularly to complex aspects that seem to not be considered during their formulation. Because of missing understanding of the complexity involved in the practice and multiple factors related to it, CE regulatory instruments have been even negatively affecting the implementation and the adoption of CE practices. Besides the category of SD Frameworks, areas of influence represented in the field of Intervenient factors can enhance such necessary understanding, when processes are analysed thorough elements indicated in the Determinants clustered factors. In the considerations developed by these CE experts, however, influences associated with the field of competences (Awareness & Knowledge-related factors) require crucial attention. This might be, for instance, a field to be intensively developed in all phases, which, according to views of this study’s participants, is definitely underdeveloped in the assessment efforts. Lack of understanding by staff in most companies and further relevant organisations about “what Circular Economy is, means, and how it should be integrated in our lives” represents a major factor hindering its comprehensive adoption. Education and training initiatives, in all levels and for all kinds of stakeholders, disseminating circularity concepts and approaches, should be a priority if this central hindering factor were to be proper addressed. From this study’s perspective, educational efforts should aim at providing more complex ways of thinking.

Aligned with this commitment, this study (i) sought to clarify less noticeable or unexplored views of aspects involved in CE adoption processes, and (ii) unearthed perspectives on roles of factors related to these processes, which facilitate the perception of their multidimensional complexity, when examining their influences. Section  Perspectives on complex influences exerted by factors highlighted conflicting effects observed simultaneously from influences exerted by one same factor, as well as nonlinear (sometimes even reticular and pervasive) influential features of this factor’s contributions to CE phenomena. Although outside the scope of this paper, such features characterise, respectively, aspects of the dialogic and the recursive principles proposed by Morin. Further in-depth examination of such characteristics of the influences observed for the factors identified shall help clarify additional complex influences of factors on the processes. These types of analysis will constitute objectives for theoretical contributions to be explored in future publications.

Future complementary recursive examination of the proposed concepts will improve the current results of the study, and their employment in the analysis of additional phenomena is intended, aiming at clarifying their limitations and identifying improvements. Deeper analyses of the identified relationships, also through comparison with theoretical perspectives for the analyses of complex interactions and social phenomena are expected to contribute to the refinement of the presented propositions. Therefore, new perspectives on the influences of factors and their fields of analysis will come to light in the next phases of this research. However, according to what has been observed in the comprehension of complex issues in this field of study, a definitive theoretical framework, which shall provide a complete view of all aspects to be considered, is seen as an unattainable goal to be reached in this study.

As previously mentioned, the purpose of this study’s proposition is not to be comprehensive in terms of the factors listed and the categories, but to develop basic concepts from the analyses of their influences that help identify the categories and the potential fields they represent, facilitating the understanding of CE phenomena. The conceptual perspectives presented are not intended to replace, or surpass, or even amend the explanatory capacity of analogous studies. They serve only the purpose of offering complementary views into influential elements in processes of adoption of CE practices. Given the complexity inherent in sustainability and CE topics and activities under analysis, a useful way to improve competences to comprehend their intricacies and complex aspects is to exercise the use of varied complementary perspectives. That is the aim of this study’s contribution.

Grounded in learnings from Morin’s epistemology of Complexity, our understanding of ‘complexifying’ views -- despite being opposed to reductionistic simplifications of reality, and against simplistic notions --, does not equal complicating perspectives on phenomena, rather promoting better comprehension of them, which corresponds to clearer understanding. Adhering to this logic, this study’s propositions strived to offer uncomplicated ways of examining its topic, from methodological choices to theoretical formulations and their representations. They may add value to the works being undertaken by scientists pursuing ways through which the complex facets of sustainability issues become understandable, and so, improving our chances of dealing successfully with the challenges of this vital area of studies.

Sustainability invites us to acquire multidisciplinary competences considered necessary to advance its science. In spite of this compelling invitation, we usually see ourselves drifted by our traditional education and professional training into insisting on disciplinary expression of our skills, knowledge, and scientific efforts. Overcoming this trend constitutes a challenge of significant proportions. Nevertheless, theoretical contributions as the ones [11–13] examined in this paper’s Introduction attest to the scientific efforts to expand the analytical capacity of concepts and approaches to investigate CE phenomena, and their propositions advance correspondingly towards increasingly complex formulations. For CE initiatives to successfully thrive, its field may require complementary efforts into establishing capacity for addressing the inherent complexity involved in the circularity’s encompassing interrelationships and implications for society’s practices.

## Data Availability

The raw data are not publicly available due to confidentiality agreements, ethical restrictions, and the risk of participant re-identification. Metadata and supporting documentation are available at https://doi.org/10.5281/zenodo.22539077.
